# 2-(5-Bromo-3-methyl­sulfinyl-1-benzofuran-2-yl)acetic acid

**DOI:** 10.1107/S1600536809008101

**Published:** 2009-03-11

**Authors:** Hong Dae Choi, Pil Ja Seo, Byeng Wha Son, Uk Lee

**Affiliations:** a Department of Chemistry, Dongeui University, San 24 Kaya-dong Busanjin-gu, Busan 614-714, Republic of Korea; b Department of Chemistry, Pukyong National University, 599-1 Daeyeon 3-dong Nam-gu, Busan 608-737, Republic of Korea

## Abstract

In the title compound, C_11_H_9_BrO_4_S, the O atom and the methyl group of the methyl­sulfinyl substituent lie on opposite sides of the plane of the benzofuran fragment. The crystal structure is stabilized by inter­molecular O—H⋯O and C—H⋯O inter­actions. In addition, the crystal structure exhibits a Br⋯π inter­action of 3.551 (3) Å between the Br atom and the centroid of the benzene ring of an adjacent mol­ecule.

## Related literature

For the crystal structures of similar 2-(5-bromo-3-methyl­sulfinyl-1-benzofuran-2-yl)acetic acid derivatives, see: Choi *et al.* (2008 ▶, 2009 ▶).
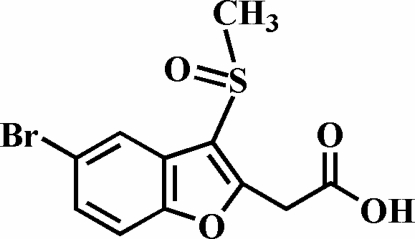


## Experimental

### 

#### Crystal data

C_11_H_9_BrO_4_S
*M* *_r_* = 317.15
Orthorhombic, 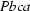

*a* = 7.7646 (7) Å
*b* = 16.304 (2) Å
*c* = 18.993 (2) Å
*V* = 2404.4 (4) Å^3^
*Z* = 8
Mo *K*α radiation
μ = 3.59 mm^−1^
*T* = 298 K
0.60 × 0.60 × 0.40 mm

#### Data collection

Bruker SMART CCD diffractometer
Absorption correction: multi-scan (*SADABS*; Sheldrick, 1999 ▶) *T*_min_ = 0.130, *T*_max_ = 0.233
13628 measured reflections
2621 independent reflections
2033 reflections with *I* > 2σ(*I*)
*R*_int_ = 0.030

#### Refinement

*R*[*F* ^2^ > 2σ(*F* ^2^)] = 0.036
*wR*(*F* ^2^) = 0.094
*S* = 1.05
2621 reflections
160 parameters
H atoms treated by a mixture of independent and constrained refinement
Δρ_max_ = 0.36 e Å^−3^
Δρ_min_ = −0.85 e Å^−3^

### 

Data collection: *SMART* (Bruker, 2001 ▶); cell refinement: *SAINT* (Bruker, 2001 ▶); data reduction: *SAINT*; program(s) used to solve structure: *SHELXS97* (Sheldrick, 2008 ▶); program(s) used to refine structure: *SHELXL97* (Sheldrick, 2008 ▶); molecular graphics: *ORTEP-3* (Farrugia, 1997 ▶) and *DIAMOND* (Brandenburg, 1998 ▶); software used to prepare material for publication: *SHELXL97*.

## Supplementary Material

Crystal structure: contains datablocks global, I. DOI: 10.1107/S1600536809008101/zl2184sup1.cif

Structure factors: contains datablocks I. DOI: 10.1107/S1600536809008101/zl2184Isup2.hkl

Additional supplementary materials:  crystallographic information; 3D view; checkCIF report

## Figures and Tables

**Table 1:** Hydrogen-bond geometry (Å, °)

*D*—H⋯*A*	*D*—H	H⋯*A*	*D*⋯*A*	*D*—H⋯*A*
O2—H2*A*⋯O4^i^	0.76 (4)	1.83 (4)	2.579 (3)	174 (4)
C5—H5⋯O4^ii^	0.93	2.62	3.453 (3)	150
C6—H6⋯O2^iii^	0.93	2.68	3.444 (4)	140

Symmetry codes: (i) 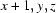; (ii) 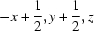; (iii) 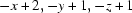.

**Fig. 1.**
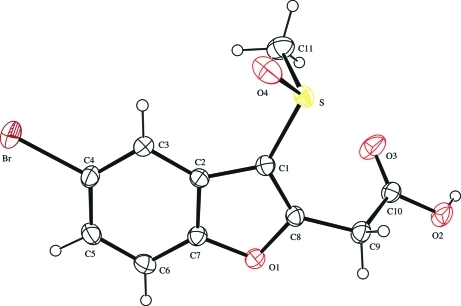
The molecular structure of the title compound, showing displacement ellipsoids drawn at the 30% probability level.

**Fig. 2.**
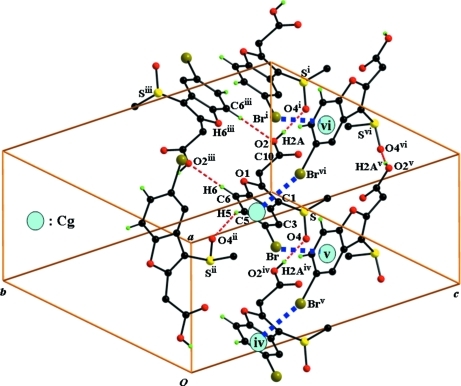
O—H···O, C–H···O and Br···π interactions (dotted lines) in the title compound. Cg denotes the ring centroid. [Symmetry codes: (i) x + 1, y, z; (ii) -x + 1/2, y+1/2, z; (iii) -x + 2, -y + 1, -z + 1; (iv) x - 1, y, z; (v) x - 1/2, y, -z + 3/2; (iv) x + 1/2, y, -z + 3/2.]

## supplementary crystallographic information

### Comment

This work is related our previous communications on the synthesis and structure of 2-(5-bromo-3-methylsulfinyl-1-benzofuran-2-yl)acetic acid derivatives, *viz*. methyl 2-(5-bromo-3-methylsulfinyl-1-benzofuran-2-yl)acetate (Choi *et al.*, 2008) and propyl 2-(5-bromo-3-methylsulfinyl-1-benzofuran-2-yl)acetate (Choi *et al.*, 2009). Here we report the crystal structure of the title compound, 2-(5-bromo-3-methylsulfinyl-1-benzofuran-2-yl)acetic acid (Fig. 1).

The benzofuran unit is essentially planar, with a mean deviation of 0.014 (2) Å from the least-squares plane defined by the nine constituent atoms. The molecular packing (Fig. 2) is stabilized by intermolecular O—H···O and C—H···O hydrogen bonds; one between the H atom of carboxyl group and the O atom of the S═O unit, a second between a benzene—H atom and the S═O unit, a third between a benzene—H atom and the hydroxy group, respectively (Fig. 2 and Table 1; symmetry code as in Fig. 2). Further stability comes from intermolecular C—Br···π interactions between the Br atom and the benzene ring of an adjacent molecule, with a C4—Br···*Cg*^v^ separation of 3.551 (3) Å (Fig. 2; *Cg* is the centroid of the C2–C7 benzene ring, symmetry code as in Fig. 2).

### Experimental

Ethyl 2-(5-bromo-3-methylsulfinyl-1-benzofuran-2-yl)acetate (276 mg, 0.8 mmol) was added to a solution of potassium hydroxide (180 mg, 3.2 mmol) in water (15 ml) and methanol (15 ml), and the mixture was refluxed for 5 h, then cooled. Water was added, and the solution was extracted with dichloromethane. The aqueous layer was acidified to pH 1 with concentrated hydrochloric acid and then extracted with chloroform, dried over magnesium sulfate, filtered and concentrated under vacuum. The residue was purified by column chromatography (ethanol) to afford the title compound as a colorless solid [yield 81%, m.p. 472–473 K; *R*_f_ = 0.36 (ethanol)]. Single crystals suitable for X-ray diffraction were prepared by evaporation of a solution of the title compound in methanol at room temperature. Spectroscopic analysis: ^1^H NMR (CDCl_3_, 400 MHz) δ 3.07 (s, 3H), 4.08 (s, 2H), 7.38 (d, J = 8.6 Hz, 1H), 7.43 (dd, J = 8.6 Hz and J = 1.82 Hz, 1H), 7.80 (d, J = 2.2 Hz, 1H), 10.54 (s, 1H); EI—MS 318 [*M*+2], 316 [*M*^+^].

### Refinement

Atom H2A of the hydroxy group was found in a difference Fourier map and refined freely. The other H atoms were positioned geometrically and refined using a riding model, with C—H = 0.93 Å for aromatic H atoms, 0.97 Å for methylene H atoms and 0.96 Å for methyl H atoms, respectively, and with *U*_iso_(H) = 1.2Ueq(C) for aromatic and methylene H atoms and 1.5Ueq(C) for methyl H atoms.

### Crystal data

**Table:**

C_11_H_9_BrO_4_S	*F*(000) = 1264
*M**_r_* = 317.15	*D*_x_ = 1.752 Mg m^−^^3^
Orthorhombic, *P**b**c**a*	Mo *K*α radiation, λ = 0.71073 Å
Hall symbol: -P 2ac 2ab	Cell parameters from 4375 reflections
*a* = 7.7646 (7) Å	θ = 2.5–26.9°
*b* = 16.304 (2) Å	µ = 3.59 mm^−^^1^
*c* = 18.993 (2) Å	*T* = 298 K
*V* = 2404.4 (4) Å^3^	Block, colorless
*Z* = 8	0.60 × 0.60 × 0.40 mm

### Data collection

**Table:**

Bruker SMART CCD diffractometer	2621 independent reflections
Radiation source: fine-focus sealed tube	2033 reflections with *I* > 2σ(*I*)
graphite	*R*_int_ = 0.030
Detector resolution: 10.0 pixels mm^-1^	θ_max_ = 27.0°, θ_min_ = 2.1°
φ and ω scans	*h* = −9→9
Absorption correction: multi-scan (*SADABS*; Sheldrick, 1999)	*k* = −17→20
*T*_min_ = 0.130, *T*_max_ = 0.233	*l* = −22→24
13628 measured reflections	

### Refinement

**Table:**

Refinement on *F*^2^	Primary atom site location: structure-invariant direct methods
Least-squares matrix: full	Secondary atom site location: difference Fourier map
*R*[*F*^2^ > 2σ(*F*^2^)] = 0.036	Hydrogen site location: difference Fourier map
*wR*(*F*^2^) = 0.094	H atoms treated by a mixture of independent and constrained refinement
*S* = 1.05	*w* = 1/[σ^2^(*F*_o_^2^) + (0.0414*P*)^2^ + 2.2996*P*] where *P* = (*F*_o_^2^ + 2*F*_c_^2^)/3
2621 reflections	(Δ/σ)_max_ = 0.001
160 parameters	Δρ_max_ = 0.36 e Å^−^^3^
0 restraints	Δρ_min_ = −0.85 e Å^−^^3^

### Special details

**Table:**

Geometry. All e.s.d.'s (except the e.s.d. in the dihedral angle between two l.s. planes) are estimated using the full covariance matrix. The cell e.s.d.'s are taken into account individually in the estimation of e.s.d.'s in distances, angles and torsion angles; correlations between e.s.d.'s in cell parameters are only used when they are defined by crystal symmetry. An approximate (isotropic) treatment of cell e.s.d.'s is used for estimating e.s.d.'s involving l.s. planes.
Refinement. Refinement of *F*^2^ against ALL reflections. The weighted *R*-factor *wR* and goodness of fit *S* are based on *F*^2^, conventional *R*-factors *R* are based on *F*, with *F* set to zero for negative *F*^2^. The threshold expression of *F*^2^ > σ(*F*^2^) is used only for calculating *R*-factors(gt) *etc*. and is not relevant to the choice of reflections for refinement. *R*-factors based on *F*^2^ are statistically about twice as large as those based on *F*, and *R*- factors based on ALL data will be even larger.

### Fractional atomic coordinates and isotropic or equivalent isotropic displacement parameters (Å^2^)

**Table:**

	*x*	*y*	*z*	*U*_iso_*/*U*_eq_	
Br	0.09253 (6)	0.55548 (3)	0.70463 (2)	0.07726 (18)	
S	0.61529 (8)	0.28188 (4)	0.61833 (4)	0.03288 (16)	
O1	0.7445 (2)	0.49916 (11)	0.54672 (9)	0.0373 (4)	
O2	1.1855 (3)	0.32375 (15)	0.53576 (12)	0.0489 (6)	
H2A	1.252 (5)	0.306 (2)	0.5603 (19)	0.057 (12)*	
O3	1.0177 (3)	0.32808 (17)	0.62986 (11)	0.0634 (7)	
O4	0.4277 (2)	0.26028 (12)	0.61107 (13)	0.0511 (6)	
C1	0.6340 (3)	0.38564 (15)	0.59570 (13)	0.0296 (5)	
C2	0.5212 (3)	0.45323 (15)	0.61355 (13)	0.0290 (5)	
C3	0.3678 (3)	0.46237 (16)	0.65037 (14)	0.0340 (6)	
H3	0.3126	0.4179	0.6711	0.041*	
C4	0.3016 (4)	0.54037 (17)	0.65473 (14)	0.0380 (6)	
C5	0.3810 (4)	0.60866 (17)	0.62502 (14)	0.0402 (6)	
H5	0.3322	0.6603	0.6306	0.048*	
C6	0.5312 (4)	0.60005 (17)	0.58753 (15)	0.0398 (6)	
H6	0.5857	0.6446	0.5667	0.048*	
C7	0.5968 (3)	0.52154 (17)	0.58250 (14)	0.0328 (5)	
C8	0.7617 (3)	0.41652 (16)	0.55538 (13)	0.0321 (6)	
C9	0.9129 (3)	0.37869 (18)	0.51972 (15)	0.0386 (6)	
H9A	0.9696	0.4202	0.4914	0.046*	
H9B	0.8723	0.3362	0.4881	0.046*	
C10	1.0426 (3)	0.34198 (16)	0.56920 (14)	0.0343 (6)	
C11	0.6508 (4)	0.2897 (2)	0.71079 (15)	0.0524 (8)	
H11A	0.5754	0.3305	0.7302	0.079*	
H11B	0.7683	0.3050	0.7194	0.079*	
H11C	0.6278	0.2377	0.7326	0.079*	

### Atomic displacement parameters (Å^2^)

**Table:**

	*U*^11^	*U*^22^	*U*^33^	*U*^12^	*U*^13^	*U*^23^
Br	0.0694 (3)	0.0723 (3)	0.0901 (3)	0.0299 (2)	0.0476 (2)	0.0178 (2)
S	0.0288 (3)	0.0292 (3)	0.0406 (4)	0.0017 (3)	−0.0038 (3)	0.0007 (3)
O1	0.0305 (9)	0.0365 (10)	0.0447 (10)	−0.0004 (8)	0.0068 (8)	0.0059 (8)
O2	0.0321 (11)	0.0708 (15)	0.0437 (12)	0.0153 (10)	0.0048 (10)	0.0039 (11)
O3	0.0390 (12)	0.109 (2)	0.0421 (13)	0.0163 (13)	0.0062 (10)	0.0189 (12)
O4	0.0328 (11)	0.0399 (11)	0.0808 (16)	−0.0076 (9)	−0.0157 (10)	0.0114 (11)
C1	0.0264 (12)	0.0309 (13)	0.0315 (12)	−0.0004 (10)	−0.0016 (10)	−0.0007 (10)
C2	0.0292 (12)	0.0289 (12)	0.0288 (12)	0.0004 (10)	−0.0018 (10)	0.0008 (10)
C3	0.0318 (14)	0.0356 (14)	0.0346 (14)	0.0005 (11)	0.0045 (11)	0.0029 (11)
C4	0.0351 (15)	0.0472 (17)	0.0317 (14)	0.0101 (12)	0.0068 (11)	0.0007 (12)
C5	0.0464 (16)	0.0339 (14)	0.0404 (15)	0.0110 (12)	0.0005 (12)	0.0010 (12)
C6	0.0443 (16)	0.0309 (14)	0.0443 (16)	−0.0001 (12)	0.0012 (13)	0.0062 (12)
C7	0.0284 (13)	0.0365 (14)	0.0334 (13)	−0.0001 (11)	0.0013 (10)	0.0016 (11)
C8	0.0289 (13)	0.0342 (13)	0.0333 (14)	0.0047 (10)	−0.0007 (10)	0.0006 (10)
C9	0.0325 (14)	0.0473 (16)	0.0360 (14)	0.0075 (12)	0.0039 (11)	0.0009 (12)
C10	0.0274 (13)	0.0380 (14)	0.0375 (15)	−0.0020 (11)	0.0023 (11)	−0.0014 (12)
C11	0.0490 (18)	0.068 (2)	0.0403 (16)	−0.0029 (16)	−0.0024 (14)	0.0116 (15)

### Geometric parameters (Å, °)

**Table:**

Br—C4	1.896 (3)	C3—H3	0.9300
S—O4	1.5051 (19)	C4—C5	1.392 (4)
S—C1	1.752 (3)	C5—C6	1.374 (4)
S—C11	1.782 (3)	C5—H5	0.9300
O1—C8	1.364 (3)	C6—C7	1.381 (4)
O1—C7	1.382 (3)	C6—H6	0.9300
O2—C10	1.313 (3)	C8—C9	1.490 (3)
O2—H2A	0.76 (4)	C9—C10	1.502 (4)
O3—C10	1.190 (3)	C9—H9A	0.9700
C1—C8	1.350 (3)	C9—H9B	0.9700
C1—C2	1.448 (3)	C11—H11A	0.9600
C2—C3	1.389 (4)	C11—H11B	0.9600
C2—C7	1.391 (4)	C11—H11C	0.9600
C3—C4	1.374 (4)		
			
O4—S—C1	106.49 (12)	C7—C6—H6	121.8
O4—S—C11	104.87 (15)	C6—C7—O1	125.8 (2)
C1—S—C11	99.22 (15)	C6—C7—C2	123.9 (2)
C8—O1—C7	106.42 (19)	O1—C7—C2	110.4 (2)
C10—O2—H2A	112 (3)	C1—C8—O1	111.4 (2)
C8—C1—C2	107.1 (2)	C1—C8—C9	133.0 (3)
C8—C1—S	124.1 (2)	O1—C8—C9	115.5 (2)
C2—C1—S	128.86 (19)	C8—C9—C10	114.1 (2)
C3—C2—C7	119.3 (2)	C8—C9—H9A	108.7
C3—C2—C1	135.9 (2)	C10—C9—H9A	108.7
C7—C2—C1	104.7 (2)	C8—C9—H9B	108.7
C4—C3—C2	116.8 (2)	C10—C9—H9B	108.7
C4—C3—H3	121.6	H9A—C9—H9B	107.6
C2—C3—H3	121.6	O3—C10—O2	124.3 (3)
C3—C4—C5	123.4 (2)	O3—C10—C9	124.9 (3)
C3—C4—Br	118.1 (2)	O2—C10—C9	110.7 (2)
C5—C4—Br	118.6 (2)	S—C11—H11A	109.5
C6—C5—C4	120.3 (3)	S—C11—H11B	109.5
C6—C5—H5	119.9	H11A—C11—H11B	109.5
C4—C5—H5	119.9	S—C11—H11C	109.5
C5—C6—C7	116.4 (3)	H11A—C11—H11C	109.5
C5—C6—H6	121.8	H11B—C11—H11C	109.5
			
O4—S—C1—C8	−137.7 (2)	C8—O1—C7—C6	179.9 (3)
C11—S—C1—C8	113.7 (2)	C8—O1—C7—C2	−0.1 (3)
O4—S—C1—C2	40.9 (3)	C3—C2—C7—C6	2.0 (4)
C11—S—C1—C2	−67.7 (3)	C1—C2—C7—C6	−179.0 (3)
C8—C1—C2—C3	177.3 (3)	C3—C2—C7—O1	−178.1 (2)
S—C1—C2—C3	−1.5 (5)	C1—C2—C7—O1	0.9 (3)
C8—C1—C2—C7	−1.5 (3)	C2—C1—C8—O1	1.5 (3)
S—C1—C2—C7	179.8 (2)	S—C1—C8—O1	−179.63 (18)
C7—C2—C3—C4	−1.2 (4)	C2—C1—C8—C9	−178.5 (3)
C1—C2—C3—C4	−179.8 (3)	S—C1—C8—C9	0.4 (4)
C2—C3—C4—C5	−0.6 (4)	C7—O1—C8—C1	−0.9 (3)
C2—C3—C4—Br	−179.94 (19)	C7—O1—C8—C9	179.1 (2)
C3—C4—C5—C6	1.7 (4)	C1—C8—C9—C10	−64.2 (4)
Br—C4—C5—C6	−179.0 (2)	O1—C8—C9—C10	115.8 (3)
C4—C5—C6—C7	−0.9 (4)	C8—C9—C10—O3	14.6 (4)
C5—C6—C7—O1	179.2 (2)	C8—C9—C10—O2	−168.3 (2)
C5—C6—C7—C2	−0.9 (4)		

### Hydrogen-bond geometry (Å, °)

**Table:**

*D*—H···*A*	*D*—H	H···*A*	*D*···*A*	*D*—H···*A*
O2—H2A···O4^i^	0.76 (4)	1.83 (4)	2.579 (3)	174 (4)
C5—H5···O4^ii^	0.93	2.62	3.453 (3)	150
C6—H6···O2^iii^	0.93	2.68	3.444 (4)	140

Symmetry codes: (i) *x*+1, *y*, *z*; (ii) −*x*+1/2, *y*+1/2, *z*; (iii) −*x*+2, −*y*+1, −*z*+1.
